# A Comprehensive Study of Al_2_O_3_ Mechanical Behavior Using Density Functional Theory and Molecular Dynamics

**DOI:** 10.3390/molecules29051165

**Published:** 2024-03-05

**Authors:** Mostafa Fathalian, Eligiusz Postek, Masoud Tahani, Tomasz Sadowski

**Affiliations:** 1 Institute of Fundamental Technological Research, Polish Academy of Sciences, Pawińskiego 5B, 02-106 Warsaw, Poland; mfath@ippt.pan.pl (M.F.); epostek@ippt.pan.pl (E.P.); mtahani@ippt.pan.pl (M.T.); 2 Department of Mechanical Engineering, Ferdowsi University of Mashhad, Mashhad 9177948974, Iran; 3 Department of Solid Mechanics, Lublin University of Technology, 20-618 Lublin, Poland

**Keywords:** Al_2_O_3_, fracture toughness, density functional theory, molecular dynamics

## Abstract

This study comprehensively investigates Al_2_O_3_′s mechanical properties, focusing on fracture toughness, surface energy, Young’s modulus, and crack propagation. The density functional theory (DFT) is employed to model the vacancies in Al_2_O_3_, providing essential insights into this material’s structural stability and defect formation. The DFT simulations reveal a deep understanding of vacancy-related properties and their impact on mechanical behavior. In conjunction with molecular dynamics (MD) simulations, the fracture toughness and crack propagation in Al_2_O_3_ are explored, offering valuable information on material strength and durability. The surface energy of Al_2_O_3_ is also assessed using DFT, shedding light on its interactions with the surrounding environment. The results of this investigation highlight the significant impact of oxygen vacancies on mechanical characteristics such as ultimate strength and fracture toughness, drawing comparisons with the effects observed in the presence of aluminum vacancies. Additionally, the research underscores the validation of fracture toughness outcomes derived from both DFT and MD simulations, which align well with findings from established experimental studies. Additionally, the research underscores the validation of fracture toughness outcomes derived from DFT and MD simulations, aligning well with findings from established experimental studies. The combination of DFT and MD simulations provides a robust framework for a comprehensive understanding of Al_2_O_3′_s mechanical properties, with implications for material science and engineering applications.

## 1. Introduction

Ceramics, a class of materials characterized by their exceptional hardness, chemical stability, and thermal resistance, have played a pivotal role in various technological and industrial applications [1,2,3]. Zhao [4] systematically classified ceramic types into four distinct categories, which encompassed Al_2_O_3_-based ceramics, Si_3_N_4_-based ceramics, SiAlON-based ceramics, and cermet tool materials. Among the numerous ceramic materials, aluminum oxide, commonly known as alumina, is a prominent choice due to its remarkable properties, including high mechanical strength, electrical insulation, and biocompatibility. The high hardness, wear resistance, and outstanding mechanical properties of alumina ceramics have led to them being widely used. These unique characteristics make Al_2_O_3_ ceramics a critical material in fields ranging from aerospace engineering to medical implants. Understanding the mechanical behavior of Al_2_O_3_ at the atomic and molecular levels is essential for optimizing its performance in these applications [5,6,7].

In recent decades, the advent of computational methods, such as density functional theory and molecular dynamics simulations, has significantly enhanced our ability to explore the mechanical properties of materials with unprecedented precision [8,9]. DFT [10], a quantum mechanical approach, provides insights into the electronic structure, thermodynamics, and chemical bonding within materials. On the other hand, the MD method [11] offers a dynamic perspective by tracking the positions and velocities of atoms as they evolve.

Two fundamental mechanical properties of Al_2_O_3_ ceramics, surface energy and Young’s modulus, have been extensively studied using DFT and MD methods [12,13,14]. Surface energy, which describes the energy required to create a new surface, is crucial in understanding materials’ reactivity and adhesive properties. Young’s modulus, representing the material’s stiffness, is a fundamental mechanical property that characterizes its response to external forces and deformations. These properties are particularly interesting in the context of Al_2_O_3_ ceramics due to their importance in various applications, such as coatings, where surface interactions and mechanical stability are essential.

The resistance to crack propagation stands as a paramount characteristic among structural materials. Fracture toughness, a pivotal material property within materials science, denotes the critical stress intensity factor of a sharp crack at which the crack’s propagation transitions into a rapid and unrestricted mode I [15]. Investigating fracture toughness provides valuable insights into the failure mechanisms of materials. Analyzing how cracks are formed and propagate helps identify weak points in materials and aids in developing strategies to prevent or mitigate such failures. The parameter *K_IC_* is widely recognized within fracture mechanics models, serving as a phenomenological material descriptor necessitating experimental calibration. An in-depth comprehension of the interplay between the physical, crystallographic, and, notably, microstructural attributes and a material’s capacity to withstand failure is of paramount significance. This comprehension forms the cornerstone for enhancing the efficacy of materials and material models. The fracture toughness of Al_2_O_3_ ceramics, a measure of their resistance to crack propagation, has been the subject of significant research. Griffith’s theory [16], developed in the early 1920s, provided a foundation for understanding fracture mechanics in brittle materials. Ohring [17] provides insights into the fracture mechanics of brittle materials, connecting critical stress, flaw size, and material properties with practical applications to coatings and their susceptibility to fracture or delamination under various conditions. Ohring’s meticulous investigations in thin films and fracture mechanics have played a pivotal role in enhancing our comprehension of fracture toughness and the intricate dynamics of ceramics’ fracture behavior, particularly within the demanding framework of applied tensile stress.

The application of DFT and MD simulations has allowed for a deeper exploration of theories, such as Griffith’s theory and Ohring’s, providing insights into the atomic-scale mechanisms governing crack initiation and propagation within Al_2_O_3_ ceramics. By considering the energy landscapes and stress distributions within the material, researchers can better understand how cracks propagate and influence the overall fracture behavior. For instance, Zhou et al. [18] investigated the bilayer structure of brittle materials and analyzed interfacial crack propagation at the interface under mixed-mode loadings employing the MD method. Lazar and Podloucky [19] conducted a study on cleavage under loading mode I by utilizing DFT calculations, where the atomic layers of SiC were permitted to relax after initiating a crack with a specified opening. They obtained critical or maximum stresses for relaxed cleavage, significantly larger than those for ideal brittle cleavage.

Zhang et al. [20] studied microstructure, growth mechanism, and mechanical properties of Al_2_O_3_-based eutectic ceramic in situ composites. They determined the fracture toughness values for Al_2_O_3_/YAG/ZrO_2_ to be *K_IC_* = 8.0 ± 2.0 MPa √m, whereas for Al_2_O_3_/YAG, the calculated fracture toughness was *K_IC_* = 3.6 ± 0.4 MPa √m. Quinten and Arnold [21] investigated ultrasonic techniques to gain information on the R-curve behavior of Al_2_O_3_ ceramics. A reduction in the sound velocity was observed at the crack tip of subcritical grown cracks. Moreover, they conducted in situ experiments utilizing a scanning acoustic microscope and observed phenomena associated with the interaction of serrated crack walls. Norton et al. [22] investigated micrometer-scale fracture behavior in single-crystal, bicrystal, and polycrystalline Al_2_O_3_ using microcantilevers with notches and engineered defects created via focused ion beam (FIB) techniques. The results were influenced by ion implantation at the notch tip, moisture-assisted slow crack propagation, and finite notch tip radius. Proposed methods for mitigating or correcting these effects allow for measuring fracture toughness (*K_C_*) and the threshold stress intensity for subcritical crack growth (*K_0_*) within individual grains and grain boundaries in typical microstructures. Schlacher et al. [23] researched on the fracture resistance of textured alumina, attributing it to crack deflection along grain boundaries. In this study, the researchers quantitatively assessed and compared the micro-scale fracture toughness of textured alumina grains and grain boundaries using micro-bending tests. Their findings revealed that the micro-scale fracture toughness of the textured alumina grain boundaries (2.3 ± 0.2 MPa √m) was approximately 30% lower than that of the grains (3.3 ± 0.2 MPa √m).

More studies must simultaneously employ DFT and MD to investigate fracture behavior. In this exploration, we delve into the advances in our understanding of Al_2_O_3_ ceramics’ mechanical properties, focusing on surface energy, Young’s modulus, and fracture toughness. We utilize DFT and MD simulations to delve into the mechanical properties, particularly the fracture behavior, of Al_2_O_3_ at both atomic and molecular scales. An intriguing avenue of inquiry centers on investigating the impact of defects, notably aluminum (Al) and oxygen (O) vacancies, on the mechanical properties of Al_2_O_3_ ceramics. The presence of such vacancies exerts a notable influence on a spectrum of mechanical attributes, encompassing ultimate strength, fracture toughness, and surface energy. In order to scrutinize this phenomenon, DFT simulations were employed, affording a detailed examination of the structural implications of these vacancies on the mechanical behavior of Al_2_O_3_ ceramics, and MD simulations were performed to explore the propagation of cracks in Al_2_O_3_, alongside the determination of its fracture toughness. By shedding light on the intricacies of Al_2_O_3′_s mechanical behavior, this research enriches our fundamental knowledge and paves the way for designing enhanced materials and structures for various applications.

## 2. Simulation Results and Discussion

### 2.1. DFT Investigation of Asymmetric O and Al Vacancies in α-Al_2_O_3_

**Figure 1:**
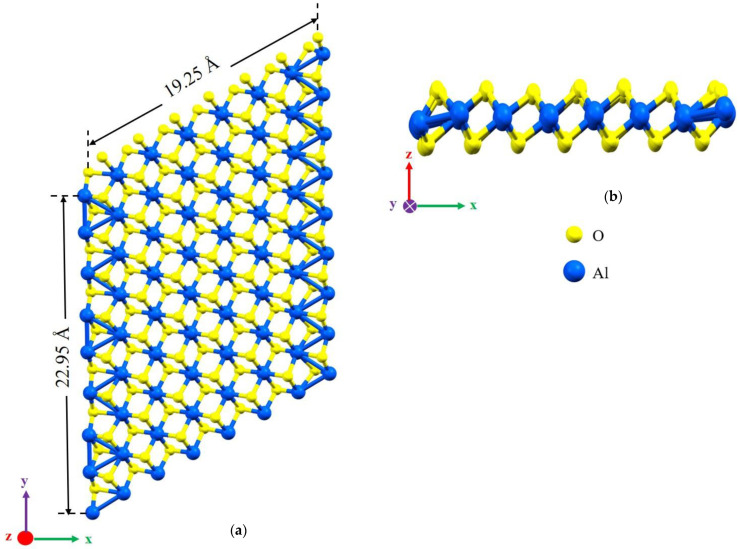
The defect-free structures of α-Al_2_O_3_ configuration after relaxation. (**a**) The top view and (**b**) the side view.

**Figure 2:**
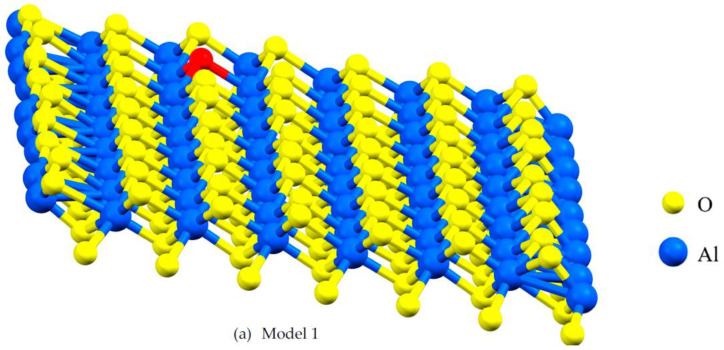

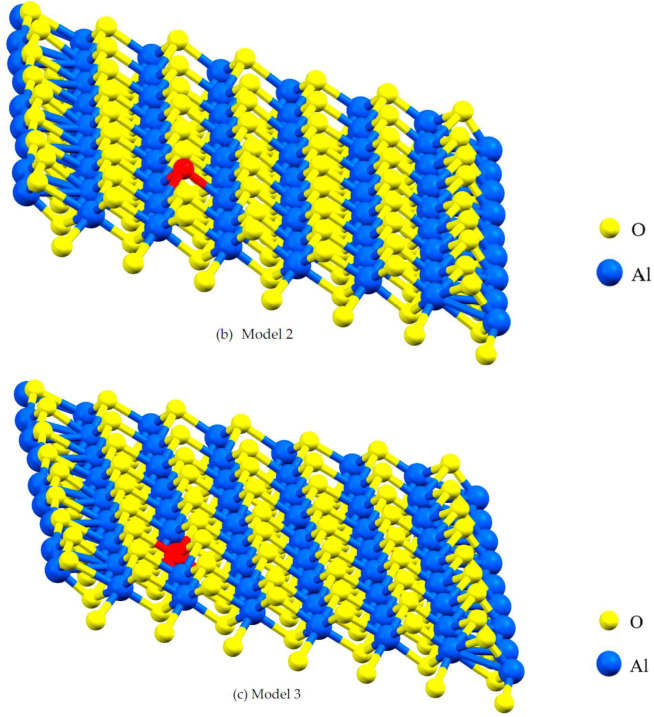

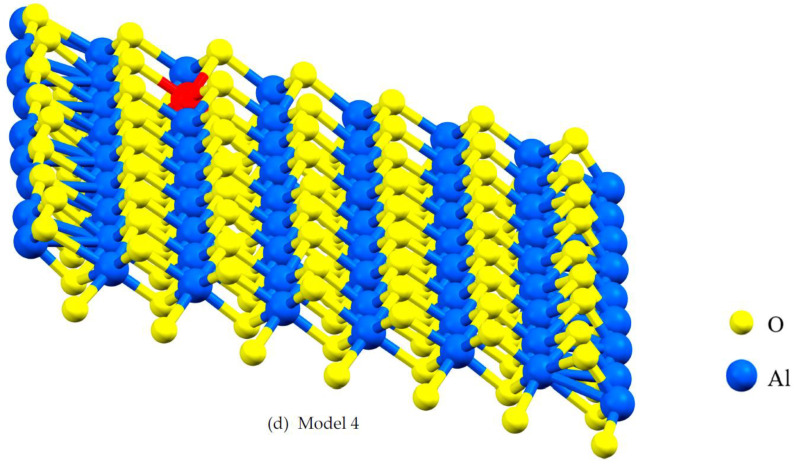
A lattice with single point vacancy by removing one atom from the corresponding layer in the α- Al_2_O_3_ configuration. The red-colored atoms represent oxygen and aluminum, both of which have been removed to create vacancies. Vacancies in Models 1 and 2 are oxygen vacancies (**a**,**b**) and vacancies in Models 3 and 4 are aluminum vacancies (**c**,**d**).

Vacancies can affect mechanical and electronic properties, and their proportion and position are critical factors in modeling with DFT. Vacancies change stress distribution and local strain fields, influencing mechanical properties such as strength, ductility, and fracture toughness. Furthermore, vacancies give rise to localized conditions within the electronic band configuration, leading to changes in electronic characteristics, including electrical conductivity and optical attributes. The percentage of vacancies significantly influences factors such as stress concentration, the initiation and spread of dislocations, deformation mechanisms, and the quantity and spatial distribution of localized electronic states within the structure [24]. This study focuses on the impact of asymmetric O and Al vacancies on the surface of α-Al_2_O_3_. To explore the properties of α-Al_2_O_3_, the initial geometric parameters of this structure were designed and optimized using DFT framework calculations, as shown in Figure 1. For the α-Al_2_O_3_ configuration, we constructed four models, each featuring distinct point vacancies and a defect-free supercell. The models, labeled Model 1, Model 2, Model 3, and Model 4, are illustrated in Figure 2 for comparison. As can be seen in Figure 2, Model 1 corresponds to the removal of one oxygen atom from the surface, Model 2 involves the removal of another oxygen atom, Model 3 involves the removal of one aluminum atom, and Model 4 entails the removal of yet another aluminum atom from the structures.

### 2.2. Formation Energy

A single vacancy formation energy can be calculated in Equation (1):

(1) $$U_{f}=(U_{v}+X_{v}-U_{T})/A$$

where $U_{v}$ represents the free energy (*eV*) of the system with a vacancy, *U_T_* is the total energy (eV) of the corresponding complete supercell without a vacancy, $X_{v}\;$ stands for the potential energy (eV) of the original atom at the vacancy site, and *A* denotes the total surface area (Å^2^). The formation energy for each vacancy position system is shown in Table 1.

Table 1 shows four models for the generation of vacancies, each characterized by a unique placement of positions. Specifically, Models 1 and 2 pertain to oxygen vacancies, while Models 3 and 4 are associated with aluminum vacancies. The observed trend in the data indicates that Models 3 and 4, involving aluminum vacancies, exhibit slightly lower formation energies (0.308 J/m^2^ and 0.306 J/m^2^, respectively) compared to Models 1 and 2, which encompass oxygen vacancies with formation energies of 0.290 J/m^2^ and 0.298 J/m^2^, respectively. Table 1 data suggest that oxygen vacancies are more energetically favorable and have lower formation energies than aluminum vacancies in α-Al_2_O_3_. These data hold significant importance, as they provide insights into the influence of formation energies on the prevalence of different types of defects in the material. They indicate that oxygen vacancies are more likely, whereas aluminum vacancies are less likely, to occur naturally due to their higher formation energies.

### 2.3. Young’s Modulus

**Figure 3:**
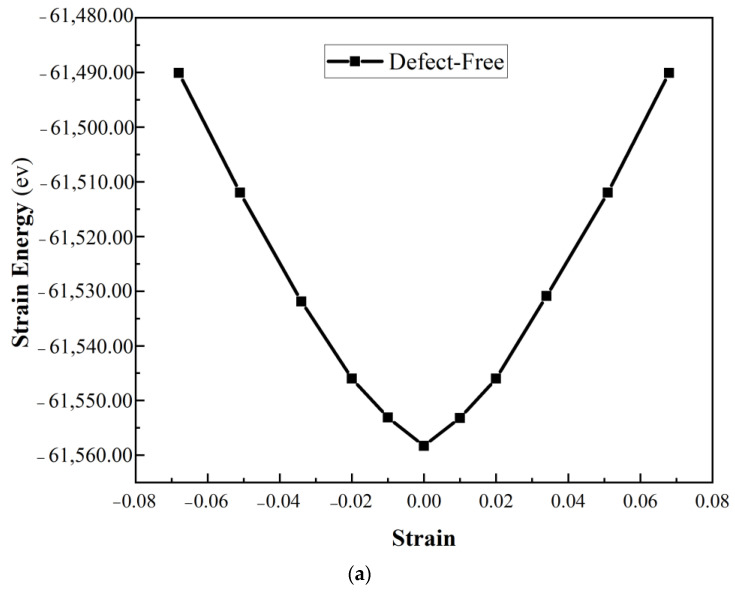

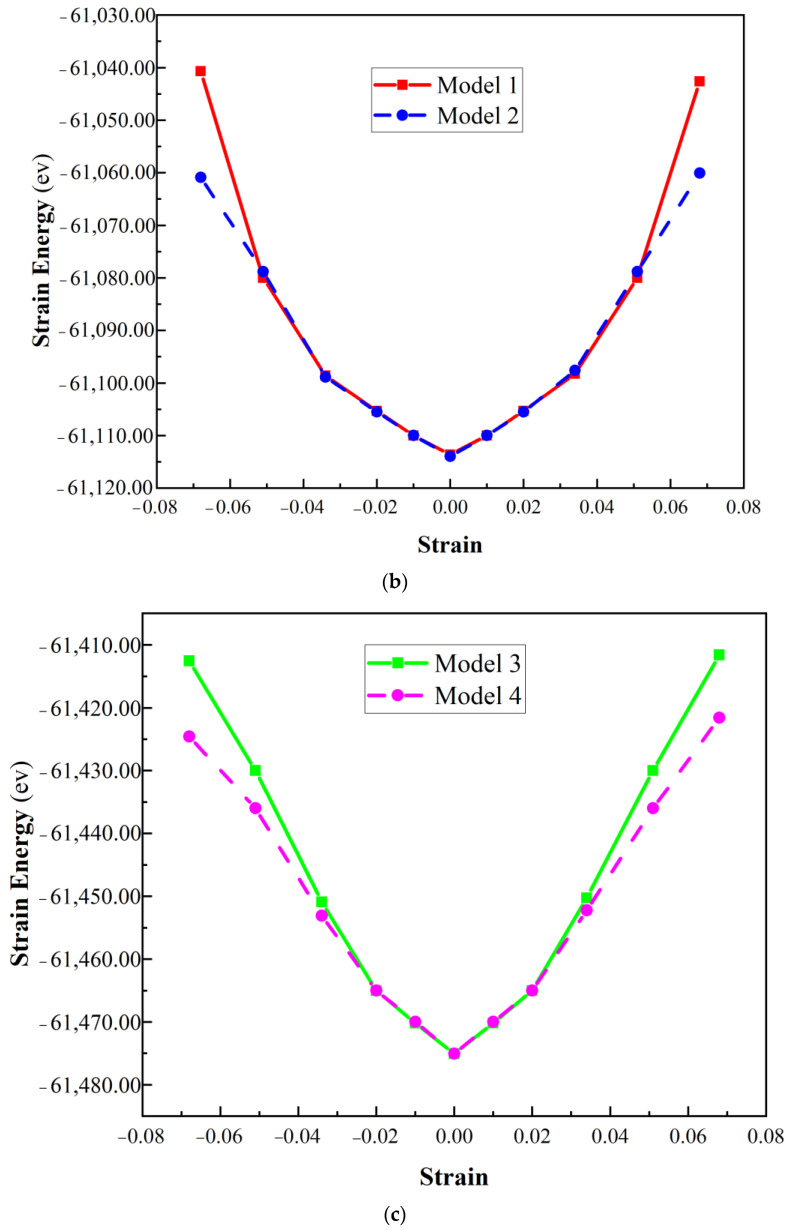
Strain energy versus strain for uniaxial strain (**a**) defect-free, (**b**) Model 1 and Model 2, (**c**) Model 3 and Model 4 of α-Al_2_O_3_ configuration.

**Figure 4:**
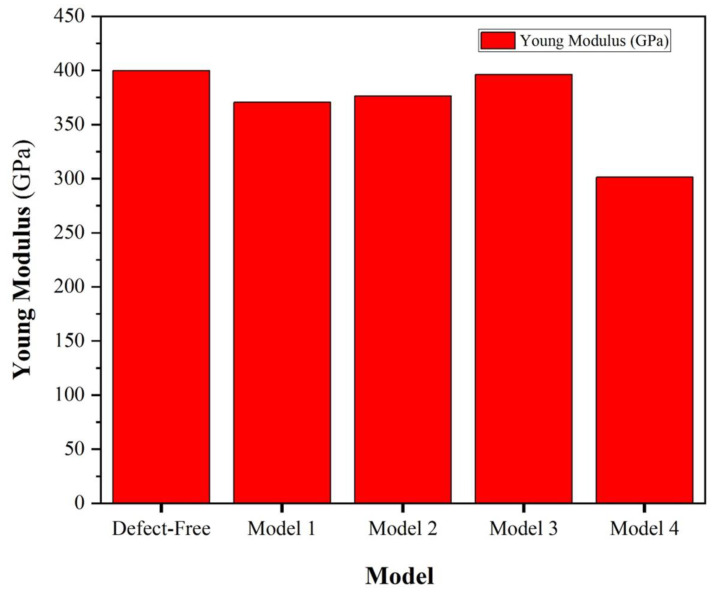
Young’s modulus for defect-free, Model 1, Model 2, Model 3, and Model 4 of α-Al_2_O_3_ configuration.

A Young’s modulus was also calculated for the α-Al_2_O_3_. Due to this, all of these alumina configurations have been compressed and stretched along the *z* direction with a small increment (1.00709 Å) [25,26,27]. Eventually, the strain energy versus strain was plotted, as shown in Figure 3. According to the following formula [28], Young’s modulus can be calculated by taking the second derivative of the total energy of the systems over the equilibrium volume.

(2) $${E=\frac{1}{V}\left(\frac{d^{2}U}{d\varepsilon^{2}}\right)}_{\varepsilon =0}$$

where *V* represents the volume of the α-Al_2_O_3_ supercell, *U* denotes potential energy, and ε is defined as Δ*L/L_0_*, where Δ*L* is the change in bulk length relative to the initial bulk length. It is essential to highlight that the total energy values mentioned have been fitted to a polynomial. The obtained Young’s modulus values for different models in α-Al_2_O_3_ present insightful information regarding the material’s mechanical behavior under various defect conditions, Figure 4. The defect-free model registers Young’s modulus of 399.83 GPa, serving as a baseline for comparison, which is very close to available experimental results [29]. The reduction in modulus observed in Model 1 (396.19 GPa) and Model 2 (376.38 GPa) associated with O vacancies suggests a slight susceptibility to decreased stiffness, potentially indicating localized structural alterations. Similarly, the diminished values in Model 3 (370.52 GPa) and Model 4 (301.3 GPa), linked to Al vacancies, imply a more pronounced impact on the material’s overall elasticity, indicative of a more significant structural disruption. The more considerable reduction in Young’s modulus in Model 4 suggests that the structural alterations resulting from Al vacancies have a more severe impact on the mechanical integrity of α-Al_2_O_3_, potentially involving a higher degree of disruption in the crystal lattice.

### 2.4. Tensile Test Simulation

A simulated tensile test investigated the tensile fracture processes of α-Al_2_O_3_. In the simulation, all atomic positions underwent gradual displacement in the *z*-direction, with incremental steps of 1.00709 Å. Throughout this process, the atoms in the entire system maintained their relative positions along the z-direction in the new configuration. Subsequently, the atomic positions of the new configuration were fully relaxed at each displacement step.

In other words, the stress–strain curves were derived by progressively deforming the simulation box in the direction of the applied displacement. Simultaneously, a relaxation of both atomic basis vectors orthogonal to the applied displacement appears. Given that the α-Al_2_O_3_ structure is represented as an atomic configuration in the DFT calculation, the stress value in each graph signifies the average stress experienced by the atoms [30,31].

**Figure 5:**
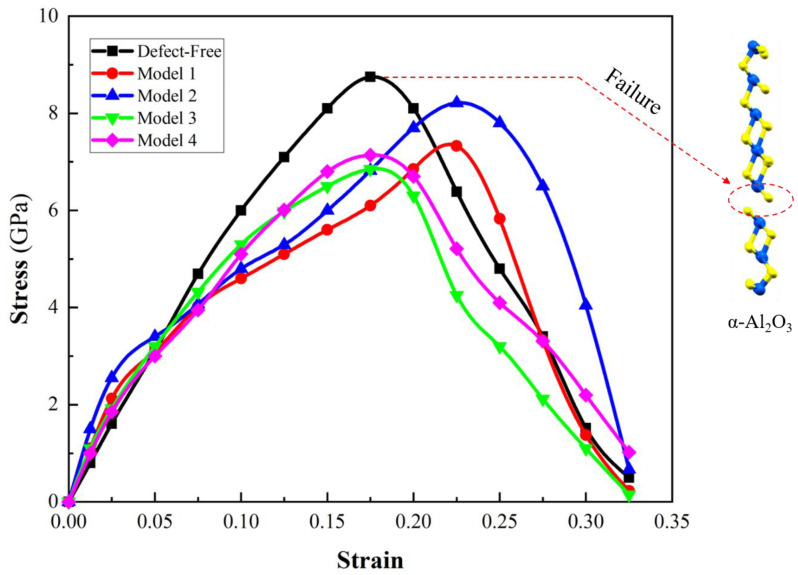
The stress-strain curves for the defect-free α-Al_2_O_3_ and α-Al_2_O_3_ with various vacancies under tensile loading.

The stress tensor, denoted as *σ_i_*_j_, is expressed in relation to the individual components of the strain tensor, *ε_ij_*, through Equation (3).

(3) $$\sigma_{ij}=\frac{1}{Ω}\left(\frac{\partial U}{{\partial \varepsilon }_{ij}}\right)$$

where *U* is the total energy and Ω represents the supercell volume [32]. Following that, the tensile stress-strain curve is obtained (Figure 5). The stress–strain curves under five distinct conditions, encompassing defect-free vacancy Models 1 and 2 (oxygen vacancy) and vacancy Models 3 and 4 (aluminum vacancy), are illustrated.

In the defect-free α-Al_2_O_3_ model, the ultimate strength is 8.75 GPa. Among all models, this ultimate strength represents the highest stress that can be withstood before failure. Beyond this point, the stress begins to decrease, indicating the initiation and progression of material failure. The specific strain at which this occurs provides critical information about the α-Al_2_O_3_ structural limits and the onset of irreversible changes in its atomic arrangement. The stress-strain profile for Model 1 implies that the presence of an oxygen vacancy at this site diminishes the ultimate strength of α-Al_2_O_3_ (7.32 GPa). In Model 2, the stress-strain response diverges from that of Model 1. The ultimate strength implies that the location of the oxygen vacancy plays a pivotal role in determining the material’s strength. Model 3′s stress-strain curve demonstrates a mechanical response that diverges from the defect-free state and oxygen vacancy models. The presence of an aluminum vacancy at position 3 imparts unique characteristics, deviating from the pristine behavior of the defect-free state and distinct effects associated with oxygen vacancies. As a result, the aluminum vacancy has a nuanced influence on the material’s deformation, highlighting its specific influence on the mechanical properties of α-Al_2_O_3_. The associated failure mechanisms also reveal a nuanced interplay, showcasing the intricate influence of the vacancy’s specific location.

In Model 4, the stress-strain curve depicts the influence of an aluminum vacancy at a specific position within the α-Al_2_O_3_ structure. Notably, the ultimate strength observed in Model 4 is close to that of Model 3, with values of 7.14 GPa and 6.85 GPa, respectively. The result suggests that the position of aluminum vacancies does not significantly influence the ultimate strength of α-Al_2_O_3_. Despite these unique conditions, the material’s resistance to failure appears to be fairly consistent, emphasizing its robust mechanical response.

### 2.5. Analysis of α-Al_2_O_3_ Surface Energy

To explore specific mechanical properties of α-Al_2_O_3_, an initial examination of its surface energy is essential. Equation (4) quantifies this (4) [33,34,35]:

(4) $$Ɣ=\frac{U_{slab}-(\frac{N_{slab}}{N_{bulk}})U_{bulk}}{2A_{slab}}$$

where *U_slab_* is the total energy of the system, *U_bulk_* is bulk energy per atom, *N_slab_* is the total number of atoms in the slab structure, and *A_Slab_* is the area of the surface unit cell. The obtained surface energy values for α-Al_2_O_3_ through DFT simulations reveal a systematic decrease as different vacancy models are introduced. Table 2 provides information about the surface energy of α-Al_2_O_3_. The defect-free model exhibits a surface energy of 8.1 J/m^2^, while oxygen vacancies (Models 1 and 2) result in surface energies of 7.1 J/m^2^ and 6.8 J/m^2^, respectively. Similarly, aluminum vacancies (Models 3 and 4) lead to 6.2 J/m^2^ and 5.9 J/m^2^ surface energies. Decreased surface energy implies that aluminium vacancies influence the material’s surface reactivity and bonding configurations. In turn, these changes in surface properties may have implications for the mechanical behavior of α-Al_2_O_3_, suggesting that introducing aluminum vacancies could impact its toughness, adhesion, or other relevant properties. The specific role of aluminum vacancies in shaping the surface energy highlights their importance in governing surface characteristics and potentially influencing the overall mechanical properties of α-Al_2_O_3_.

### 2.6. Fracture Toughness in α-Al_2_O_3_ via DFT and MD Simulations

**Figure 6:**
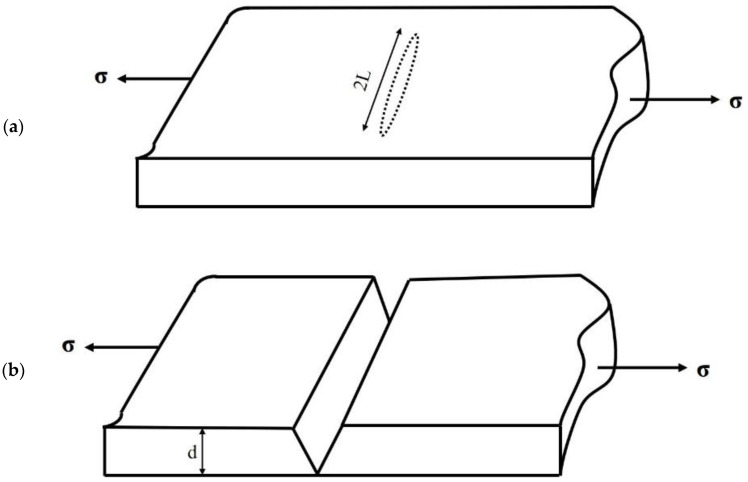
Flat elliptical crack of length 2L in a loaded uniaxial intension (**a**); Crack leading to fracture (**b**).

Regarding materials science, fracture toughness is the ability of a material containing a crack to resist fracture. Ohring’s [17] extensive body of work unfolds, offering profound insights into the multifaceted factors governing the fracture behavior of brittle materials. Ohring’s meticulous investigations of thin films and fracture mechanics have played a pivotal role in enhancing our comprehension of fracture toughness and the intricate dynamics of ceramics’ fracture behavior, particularly within the demanding framework of applied tensile stress (Figure 6).

Tensile stresses play a role in opening the crack, as illustrated in Figure 6. With a gradual increase of applied stress, additional elastic strain energy (*U_ε_*) is released. At any stress level, this energy has a magnitude of *σε/*2, where *U_ε_* is the strain energy per unit volume, representing the area under the elastic stress-strain curve. According to Hooke’s law, *σ = Eε*. Strain energy can be expressed as:

(5) $$U_{\varepsilon }\;=\sigma^{2}/2E\;$$

The elastic forces are countered by interatomic bonds, which need to be broken for the crack to extend further. At any given moment, the total energy $U_{T}$ associated with these opposing tendencies is expressed as (Figure 6):

(6) $${U_{T}\;=\;-\sigma }^{2}\;/2E(\pi L^{2}d)+4ƔLd$$

Instability arises when *dU_t_/dL =* 0, and straightforward differentiation subsequently provides a critical stress for crack propagation, as follows:

(7) $$\sigma_{C}=\sqrt{\frac{4ƔE}{\pi L}}$$

A fracture toughness *K_IC_* can be calculated using the following equation as a critical stress intensity factor [36,37]:

(8) $$K_{C}=\sqrt{4ƔE}$$

where *Ɣ* and *E* are surface energy and Young’s modulus, respectively. Their values are obtained from the DFT calculations. The values of fracture toughness for all imperfect models and the pristine model are shown in Table 3.

The defect-free model exhibits a fracture toughness of 3.56 MPa√m. As oxygen and aluminum vacancies are introduced (Models 1 to 4), there is variation in the fracture toughness values. Models 1 and 2, associated with oxygen vacancies at different positions, show fracture toughness values of 3.21 MPa√m and 3.19 MPa.√m, respectively. Meanwhile, for Models 3 and 4, which are associated with aluminum vacancies in different positions, the fracture toughness values are 3.14 MPa√m and 2.67 MPa√m, respectively. The decrease in fracture toughness with the introduction of vacancies suggests that the altered atomic arrangement influences the material’s resistance to crack propagation.

This pattern highlights how important vacancy type and position are in controlling Al_2_O_3_′s fracture toughness. Aluminum vacancies in the lattice can lead to weaker bonds, making it easier for cracks to propagate through the material. This reduction in fracture toughness may be attributed to changes in the surface energy and Young’s modulus caused by vacancy-induced structural disruptions. Conversely, by influencing charge balance and electronic structure, oxygen vacancies impact the material’s stress distribution differently, resulting in varying effects on fracture toughness. The complex interplay between surface energy, Young’s modulus, and the specific characteristics of each vacancy type contributes to the observed differences in fracture toughness values. The close agreement between the fracture toughness values obtained through our DFT computational method (approximately averaging 3.0 MPa√m) and the range of experimental results (between 3.0 and 5.0 MPa√m) [37,38,39] is encouraging. This convergence suggests that our computational approach provides accurate and reliable predictions of the material’s resistance to crack propagation. The consistency between the computational and experimental findings validates the utility of the DFT method in capturing the essential mechanical properties of Al_2_O_3_. The observed range within the experimental results may stem from factors such as variations in sample conditions, testing methods, or specific crystallographic orientations, reinforcing the importance of considering these factors in future analyses.

### 2.7. Crack Propagation and Fracture Toughness of α-Al_2_O_3_ via MD Simulations

**Figure 7:**
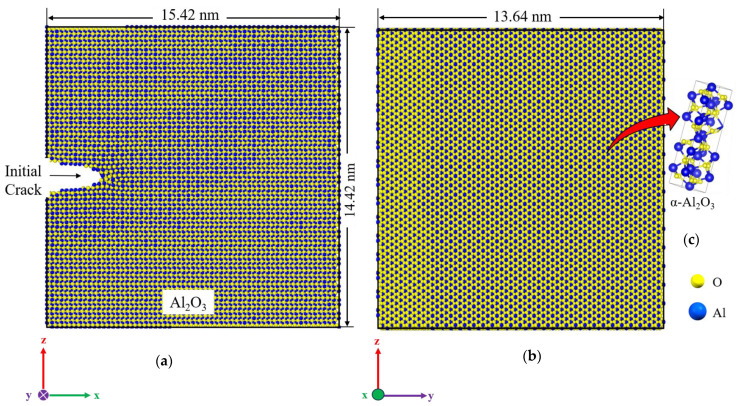
Schematic representation of the α-Al_2_O_3_ simulation model, dimensions, and coordinate system; front view, normal to *x-z* plane (**a**), thickness of the simulation box, *y-z* plane view (**b**), crystalline form of aluminum oxide, α-Al_2_O_3_ (**c**).

We additionally determined the fracture toughness of α-Al_2_O_3_ using MD simulations to validate our DFT results. Figure 7 illustrates the initial simulation box of Al_2_O_3_ prior to fracture and crack propagation for MD analysis. These configurations were obtained after undergoing the relaxation procedure at room temperature.

For mechanical testing, a crack of 29 Å is initiated at the edge, extending throughout the entire thickness of the sample, as depicted in Figure 7. The sample undergoes uniform loading in the z-direction at 300 K with a strain rate of 10^9^ s^−1^, while the other two dimensions are periodic.

Following Griffith’s theory, the total energy released in the presence of a crack can be obtained using the following equation, where the energy required to cause fracture (*Gc*) is a function of the stress (*σ*), crack length (*L*), and the elastic modulus (*E*).

(9) $$G_{c}=\frac{\sigma^{2}\pi L}{E}$$

Moreover, the association between the stress intensity factor (*K_I_*) and the energy release rate (*G*) can be expressed as follows:

(10) $$G_{c}=\frac{{K_{I}}^{2}}{E}$$

As a consequence, fracture toughness can be determined using the following formula:

(11) $$K_{IC}=\sigma \sqrt{\pi L}$$

According to the MD simulation results, the maximum stress value reached 29.79 GPa, which is critical stress for crack growth. The MD simulation reveals a fracture toughness of 2.8 MPa·√m for Al_2_O_3_, showing acceptable proximity to the fracture toughness determined in our DFT calculations (an approximate average of 3.0 MPa√m) and experimentally available data (ranging between 3.0 and 5.0 MPa√m) [38,39,40]. This consistency is illustrated in Table 4.

**Figure 8:**
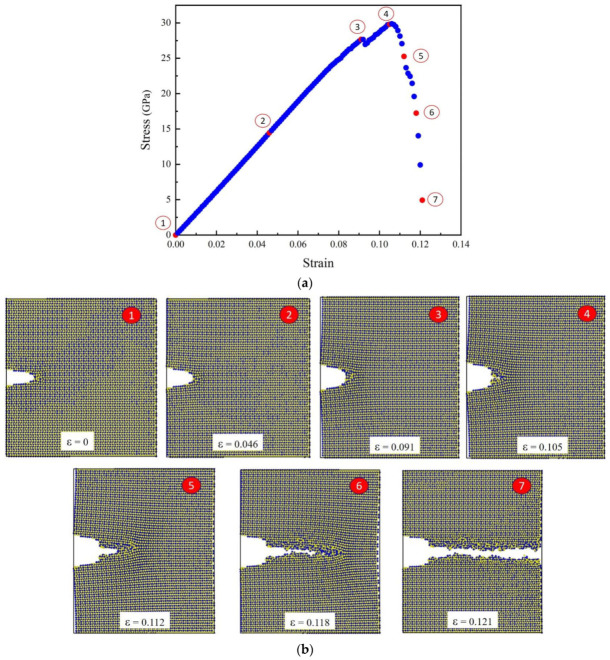
Behaviour of the sample under loading; Stress-strain curve of α-Al_2_O_3_ following tensile testing in the z-direction conducted utilizing MD (**a**), The snapshots depicting the progressive crack propagation (mode I) in Al_2_O_3_ at various strain levels (**b**).

The crack growth can be seen in several displacement snapshots, as depicted in Figure 8. Demonstration of crack propagation can play a pivotal role in deepening our understanding of how materials behave at the nanoscale:

1. It provides a visual representation of crack growth in response to displacement. This visual aid can allow us to observe the evolution of the crack directly, offering a more intuitive understanding than descriptions alone.
2. It can validate findings and demonstrate the accuracy of their simulation results. Visual evidence of crack growth serves as a means of verifying the credibility of the study.
3. It can serve as a foundation for quantitative analysis.
4. It can furnish information on crack lengths, facilitate an examination of crack propagation rates, and enable an exploration of the correlation between external factors and crack growth.

Figure 8 depicts the stress-strain curve of α- Al_2_O_3_ after conducting tensile testing in the z-direction using MD. As evident from the stress-strain curve, seven distinct points corresponding to positions 1 through 7 are discernible. At Point 1, the initial crack in the box is observed, corresponding to zero strain. Points 2 and 3 correspond to elongations of ε=0.046 and ε=0.091, respectively. As can be seen from snapshots 2 and 3, crack propagation has not yet commenced. At Point 4, the stress reaches its maximum value of 29.79 GPa, marking a critical stress for crack growth. Subsequently, shortly after Point 4, crack growth initiates in the crack tip area.

The initiation of a crack often occurs at locations of stress concentration, such as the tips of pre-existing defects within the material. These stress concentration sites are vulnerable areas where the material is more prone to failure. When the applied stress surpasses the critical strength, the material undergoes a process known as nucleation [41], where atomic bonds start to break, creating a small crack. It is evident that the stress near the crack tip gradually increases as the loading continues at the initial stage. When the stress reaches the critical value, the atoms surrounding the crack tip start to separate, indicating the initiation of a crack. Subsequently, cracking begins and increases steadily. At Points 5 and 6, the elongation is ε=0.112 and ε=0.118, respectively, while the crack propagates horizontally with an increase in displacement. Ultimately, at Point 7, the stress gradually diminishes until the propagation stops, and the crack reaches the end of its path, leading to failure. 

The stress in the material gradually decreases in this region due to the redistribution of forces and the dissipation of energy associated with crack propagation. Simultaneously, the elongation rate reaches its maximum as the material undergoes significant deformation.

## 3. Simulation Methodology

The simulation can be divided into two parts: first, DFT simulations, and second, simulation for MD calculations. DFT and MD simulations consider Alumina with a hexagonal crystal structure (corundum).

### 3.1. Density Functional Theory Models

In this work, the atomic geometry and electronic structure of α-Al_2_O_3_ were calculated by the DFT framework [42,43]. The calculations were performed using the Spanish Initiative for Electronic Simulations with Thousands of Atoms (SIESTA) code [44,45,46]. We used the generalized-gradient approximation (GGA) function with the Perdew–Burke–Ernzerhof (PBE) [47,48] to treat the effects of correlation and electronic exchange. All atomic orbital basis sets are double-ξ plus polarization orbitals (DPZ) with a 50 MeV energy shift, and the split norm was 0.3. A [5 × 5 × 1] Monkhorst–Pack grid [49,50] was used for the k-point sampling of the Brillouin zone, and the atomic locations were relaxed until the remaining forces on any atom were smaller than 0.02 eVÅ^−1^. The cutoff of the plane-wave kinetic energy is 120 Ry in the calculations. The ground state of the electrons can be found by solving the Kohn–Sham equation [42]. Periodic boundary conditions were used in all directions. Periodic boundary conditions were used with 8× 8 × 1 supercells. The vacuum height (15 Å) was set to eliminate spurious interactions between periodically repeated images. The generated samples were all fully relaxed in three directions before performing stress–strain calculations (Figure 1).

### 3.2. Molecular Dynamics Model

The lattice parameters for hexagonal α- Al_2_O_3_ are *a = b* = 4.805 Å, *c =* 13.116 Å, *α = β =* 90°, and *γ =* 120°. MD simulations were conducted using the open-source program large-scale atomic/molecular massively parallel simulator (LAMMPS) package [51]. In order to visualize the evolution of the atomic structure, the open visualization tool OVITO [52] was utilized. Newton’s second law is used to obtain the kinematic parameters of particles using the molecular dynamics method. Therefore, the atomistic model should incorporate appropriate potential functions representing atomic interactions. An ab initio calculation or experimental data were used to determine the parameters of a potential function. The COMB3 [53] potential was utilized for system relaxation, and the simulation box dimensions were set at 15.42 nm × 13.64 nm × 14.42 nm. The slab orientations were utilized along the X (100), Y (010), and Z (001) directions. The configuration’s boundary conditions were defined as non-periodic and shrink-wrapped (S) in one direction while being periodic in the other two directions. In this simulation, the relaxation of the simulation box was done within two steps. In the first step, the simulation box was kept at a constant temperature of 300 K in the NPT ensemble to allow the relaxation of the structure. In the second step, the isobaric-isothermal ensemble (NPT) was used for 30 ps to keep the constant temperature of 300 K and impose the pressure of 1 bar to get the initial physical state of the material. At the simulation box, a crack tip was introduced at the edge of the box (Figure 1) to facilitate crack propagation.

### 3.3. Potential Functions

The third-generation charge-optimized many-body potential (COMB3) [53] is a type of interatomic potential that can describe interactions between atoms in aluminum–oxygen systems. The COMB3 potential uses a combination of pair potentials and electron density functions to describe the atomic interactions. The potential is fitted to experimental data and ab initio calculations. The total energy per atom for the Al-O system, with a charge of *q* at position *r*, in the COMB3 potential can be expressed as [53]:

*U*_tot_ (*r*, *q*) = *U*_es_ (*q*, *r*) + *U*_short_ (*q*, *r*) + *U*_vdw_ (*r*) + *U*_corr_ (*r*) (12)

where *U_es_* denotes the energy required to create an atom’s charge, as well as the energies involved in charge–charge interactions, charge–nuclear interactions, and polarizability. Furthermore, *U_short_* is the energy of pairwise attractive and repulsive functions, *U_vdw_* is long-range van der Waals interactions, and *U_corr_* is the correction terms employed to adjust energies associated with specific angles outside the bond order terms.

## 4. Conclusions

In summary, this study delved into the influence of various vacancies on the mechanical characteristics of Al_2_O_3_ through DFT calculations. The analysis encompassed the examination of surface stability, mechanical behavior, and fracture mechanisms in the presence of four types of vacancies. Furthermore, the nature of bonding and critical parameters such as formation energy, surface energy (*γ*), and fracture toughness (*K_IC_*) were investigated.

Additionally, a detailed investigation into crack propagation in Al_2_O_3_ using MD simulation (Model 1) was conducted. This investigation also facilitated the determination of the fracture toughness of pristine Al_2_O_3_.

The main conclusions are as follows:

Generating aluminum vacancies requires more energy compared to creating oxygen vacancies. It means the likelihood of aluminum vacancies occurring in natural conditions is lower than oxygen vacancies.

- Young’s modulus experiences a significant decrease with aluminum vacancies compared to the modulus value for oxygen vacancies.
- Aluminum vacancies can significantly reduce elongation in a tensile test compared to elongation associated with oxygen vacancies.
- The fracture toughness of the pristine alumina is 3.56 MPa√m, closely aligning with existing experimental results [49,50,51]. However, the introduction of vacancies, particularly at Al vacancies in models 3 and 4, significantly reduces fracture toughness, measuring at 3.14 MPa√m and 2.67 MPa√m, respectively.
- The MD simulation yields a fracture toughness of 2.8 MPa√m for α-Al_2_O_3_, aligning acceptably with both our DFT calculations (approximate average of 3.0 MPa√m) and experimental data (ranging between 3.0 and 5.0 MPa√m), emphasizing the reliability of the simulation results.
- The visual representation of crack growth provides crucial insights into nanoscale material behavior, serving not only as a tool for direct observation but also as means to validate findings and establish a foundation for quantitative analysis, including crack lengths, propagation rates, and correlations with external factors. This comprehensive understanding enhances the significance and applicability of the study’s outcomes.

Exploring the atomic-level properties of α-Al_2_O_3_ can pose significant challenges. Therefore, utilizing DFT calculations and MD simulations can be an alternative approach to scrutinizing the impact of vacancies and crack propagation in ceramic materials. Subsequent research aims to explore diverse ceramic materials, drawing comparisons between DFT, MD simulations, and experimental results.

## Figures and Tables

**Table 1:** The formation energy of vacancies.

	Formation Energy (J/m^2^)
Defect-Free	-
Model 1	0.290
Model 2	0.298
Model 3	0.308
Model 4	0.306

**Table 2:** The calculated surface energy for α-Al_2_O_3_.

	Surface Energy (J/m^2^)
Defect-Free	8.1
Model 1	7.1
Model 2	6.8
Model 3	6.2
Model 4	5.9

**Table 3:** Fracture toughness of α-Al_2_O_3_ (DFT simulations).

	Fracture Toughness (MPa√m)
Defect-Free	3.56
Model 1	3.21
Model 2	3.19
Model 3	3.14
Model 4	2.67

**Table 4:** Fracture toughness of α-Al_2_O_3_ by DFT, MD, and experiments.

	Fracture Toughness (MP√m)
DFT	3.56
MD	2.8
Exp.	3–5 [37,38,39]

## Data Availability

Data available on request.
